# Computing patient data in the cloud: practical and legal considerations for genetics and genomics research in Europe and internationally

**DOI:** 10.1186/s13073-017-0449-6

**Published:** 2017-06-20

**Authors:** Fruzsina Molnár-Gábor, Rupert Lueck, Sergei Yakneen, Jan O. Korbel

**Affiliations:** 1 Heidelberg Academy of Sciences and Humanities, Karlstraße 4, 69117 Heidelberg, Germany; 2 European Molecular Biology Laboratory, Genome Biology Unit, Meyerhofstraße 1, 69117 Heidelberg, Germany

## Abstract

Biomedical research is becoming increasingly large-scale and international. Cloud computing enables the comprehensive integration of genomic and clinical data, and the global sharing and collaborative processing of these data within a flexibly scalable infrastructure. Clouds offer novel research opportunities in genomics, as they facilitate cohort studies to be carried out at unprecedented scale, and they enable computer processing with superior pace and throughput, allowing researchers to address questions that could not be addressed by studies using limited cohorts. A well-developed example of such research is the Pan-Cancer Analysis of Whole Genomes project, which involves the analysis of petabyte-scale genomic datasets from research centers in different locations or countries and different jurisdictions. Aside from the tremendous opportunities, there are also concerns regarding the utilization of clouds; these concerns pertain to perceived limitations in data security and protection, and the need for due consideration of the rights of patient donors and research participants. Furthermore, the increased outsourcing of information technology impedes the ability of researchers to act within the realm of existing local regulations owing to fundamental differences in the understanding of the right to data protection in various legal systems. In this Opinion article, we address the current opportunities and limitations of cloud computing and highlight the responsible use of federated and hybrid clouds that are set up between public and private partners as an adequate solution for genetics and genomics research in Europe, and under certain conditions between Europe and international partners. This approach could represent a sensible middle ground between fragmented individual solutions and a “one-size-fits-all” approach.

## Background: challenges and current solutions for pan-cancer translational genomics research

Recent decreases in the cost of genome sequencing have driven forward several large-scale initiatives in basic and translational genomics research [1–4] (see, for example, the International Cancer Genome Consortium (ICGC) [5], the Pan-Cancer Analysis of Whole Genomes (PCAWG) project [6], and the 100,000 Genomes Project [7]). It is expected that hundreds of thousands of patients’ genomes will be sequenced and analyzed in the next 3–5 years [8]. When combining genomic data with other molecular data types—such as transcriptomes, microbiomes (Box 1), and clinical information— the resulting uniquely rich dataset enables integrative analyses to be carried out at unprecedented depth and scale and facilitates new insights into molecular disease processes, thus having implications for basic research and personalized healthcare. Comparative analyses across specimens collected by individual projects of the ICGC [2] may, for example, help to uncover commonalities and differences in the development and progression of different types and subtypes of cancer [1], and may inform the development of novel diagnostic and treatment strategies. A well-developed example of collaborative data sharing and analysis is the PCAWG project of the ICGC [6], which involves petabyte-scale (Box 1) genomic datasets that have been collected across research centers from different legal systems and jurisdictions (that is, the different territories or areas of activity over which the legal authority of a court or other institution extends). In this article, we refer to this project as an example “use case” (Box 1) of large-scale data integration involving genomic data from different international cohorts.

Along with these data integration opportunities, novel challenges are emerging in relation to data processing and sharing, for example. Most individual academic research centers do not currently possess the information technology (IT) infrastructure required to securely store and jointly process thousands of whole-genome sequences and similar quantities of other data. Furthermore, differences in analytical methods and their lack of standardization mean that the results of genetic and genomic analyses from different research locations are often incomparable, which impedes data re-use and reduces the benefits for research and patients.

Cloud computing (Box 1) could help to overcome many of these difficulties by allowing the rapid sharing and standardized processing of research data in a collaborative manner (Box 2) [9]. However, efforts to comparatively analyze genomic data—for example, those from different types of cancer—have revealed further challenges related to the secure cloud-based large-scale processing and collaborative sharing and storage of research data across cohorts [1]. The goal of the PCAWG project is to identify common patterns of mutation in whole-genome sequencing data from tumors and donor-matched normal tissues from >2800 patients with cancer; this project will generate nearly 1 petabyte of data.

To meet the challenges associated with pursuing research with such large-scale data, a model of collaborative and distributed computing has been developed within the PCAWG consortium [1], and it involves different partner institutions that contribute computing centers that have localized institutional clouds as well as public cloud computing capabilities. The involvement of academic partners based in countries outside of Europe (which are hereafter referred to as third countries) and of commercial partners could facilitate the compilation of large and diverse datasets through research collaboration, and could add expertise, technical capabilities, and data-processing capacity. Analyses during the initial stages of the PCAWG project—of data from ~1000 patients with cancer—were conducted in part on commercial clouds at a relatively low price and under advantageous processing conditions (that is, for example, in terms of speed and data-processing capacity).

The quantity and diversity of patient data processed on clouds are increasing in cross-border genomic data-sharing projects. Although not all of the data (including genetic and genomic data) collected have an unambiguous connection to a particular individual, the probability of being able to identify a pseudonymized (Box 1) or anonymized sample donor—whether unintentionally or intentionally—is increasing as data quantity and data diversity increase. As an example, in the context of disease studies, information about disease diagnosis and the ethnicity and age of a patient may already be sufficient for the identification of donors in a number of cases. If the data are stored on public clouds, segregation between different data categories may thus become necessary. However, rules and regulations for data sharing and redistribution differ between data types and sometimes between jurisdictions, which prevents the development of a uniform solution for data sharing that fits all use cases in genetics and genomics research.

Various global approaches to data protection, and particularly to the understanding and regulation of the right to the protection of personal data (Box 1), can be identified as a significant barrier to cross-border genetics research. In this article, we first describe how cloud computing is used for genetics and genomics research in different countries and international projects. Second, we focus on the specific challenges arising from current regulatory solutions for processing European patient datasets, particularly regarding the responsibilities of persons and entities that control data processing (data controllers; Box 1) and those that accomplish processing on their behalf (data processors; Box 1), and for transfers to third countries. Third, we consider possible ways in which international regulatory differences in data protection could be overcome in order to ensure that individual rights and freedoms are maintained while enabling genetics and genomics research and collaboration with global partners and facilitating the freedom of scientific research. Finally, we discuss possible technological solutions that could overcome the identified regulatory challenges.

## Global cloud computing of patient data for genetics and genomics research

Cloud computing is used for two main reasons in genetics and genomics research: first, to allow large-scale genomic data processing using readily scalable, external infrastructure; and second, to allow the sharing of genomic data with collaborators via a jointly usable IT environment. In the United States (US), for example, the National Institutes of Health is promoting the deposition of research data on diverse cloud platforms, including commercial clouds, to facilitate data sharing [10]. Within Europe, the European Open Science Cloud (EOSC) pilot project and related studies (for example, the German de.NBI cloud project, http://www.denbi.de) are currently exploring the use of public and public–private cloud frameworks to promote research based on shared datasets [11].

Diverse approaches for using clouds in research result in additional challenges in terms of the interoperability (Box 1) of the analytical frameworks being used. The analysis of data from global cohorts requires that researchers either copy all of the data to a common location or develop tools that are able to operate in a globally distributed manner; the development of such tools represents a significant technical challenge, as researchers use different application programming interfaces (APIs; Box 1). This has resulted in the development of novel scientific data analysis frameworks—such as Butler [12]—that are able to operate across the gamut of globally dispersed cloud computing environments to deliver analysis results in a timely manner. An additional major challenge stems from jurisdictional differences in legislation, as well as differences in donor consent (Box 1), which may permit, limit, or prevent the distribution of genetics and genomics research data with clouds, thus leading to diversification in the use of clouds between countries and to the establishment of jurisdiction-based cloud silos. Restricting the geographic locality of data stored in the cloud to specific jurisdictions is currently not standardized, and typically involves the selection of cloud providers that are able to offer services from data centers in a particular country or region, and involves appropriate contractual arrangements. To support the efficient exchange and distribution of genetic and genomic data across clouds and jurisdictions, future research will need to focus on standardizing cloud procurement (that is, obtaining or buying cloud services from an external provider) and on determining the technical elements involved in developing a common approach to protect cloud data locality (often referred to as “geo-fencing”).

In the European Union (EU) and US, for example, there are differences in the regulation of data protection. Personal data protection aims to protect the participants of genetics and genomics research and uphold their rights and freedoms, which could be compromised depending on the use of their data (Recital 1 of [13]). The EU General Data Protection Regulation (GDPR), which came into force in 2016, aims to secure a high level of protection of personal data in all member states [14]. It is applicable to a broad range of personal data-processing activities and grants individuals with various rights in relation to numerous data categories. It provides a general and uniform protection as public and private addressees of this regulation generally fall under the same legislation [15].

In the US, aspects relating to the protection of the right to data protection have been derived from the Fourth Amendment of the Constitution, which protects individuals against “unreasonable searches and seizures” [16]. The application of this Amendment is limited to those “places, things and actions” in relation to which an individual has a “legitimate expectation of privacy”, and thus excludes data that individuals voluntarily provide to third parties (page 362 of [17]; Box 1) such as personal data that have been provided for research and for which informed consent has been obtained. The constitutional validity of a search or seizure in the case of foreign intelligence surveillance for national security purposes was formally recognized in 2008 [18]. In addition, the Fourth Amendment does not apply to foreign citizens or residents [19]. In contrast to the EU, there is no horizontal data protection legislation in the US that is applicable to both public and private addressees. By limiting governmental action only, data protection in the US is mainly understood as informational seclusion and a right to be left alone (Recital 31 of [13]), rather than the effective preservation and promotion of an individual’s capacity to make free decisions about their data.

Despite these principal differences between the EU and the US in the understanding of data protection, recent changes in regulation suggest certain moves toward harmonization. Some courts in the US have recently begun to scrutinize the broad exemption of voluntary transfer (for example, that of individual data) in light of the changing electronic and technological landscape (page 22 of [20]). In addition, the Fourth Amendment has recently been applied in a judgment that has been interpreted as creating a “right to deletion” of outdated data held by law enforcement agencies (page 140 of [21]). In parallel to this, in other countries where data protection is not historically rooted in constitutional or common law (for example, Australia, India, China, and Singapore), comprehensive statutory protection is now emerging. This international trend may hence ultimately allow for the introduction of data protection regulation with a greater emphasis on the active control of data by research participants. The move toward more comprehensive global data protection could benefit international data-sharing and multicenter research projects such as the PCAWG project.

## Processing European patient data: challenges due to current regulatory solutions

Due to this cautious worldwide trend toward attributing more rights to data subjects (that is, to the individuals who provide personal data), we will now focus on the EU regulations that are relevant in the context of the PCAWG project, which we use as an example of a project in which research participants and patient donors are encouraged to have an active role in data protection.

The GDPR of the EU will apply in the member states from 25 May 2018 [14]. Although the GDPR leaves considerable room to interpret certain legal provisions, a consideration of the general standards defined in the GDPR is crucial for the evaluation of cloud models that could be used in common European genetics and genomics research. With this in mind, the GDPR has also the declared objective of facilitating the free flow of data in the European Digital Single Market (Recital 170 of [14]). As differing regulations for data processing between member states could limit the free flow of data, the GDPR claims that it harmonizes, to a high degree, both the rights of the persons affected and the obligations of data controllers and data processors. At the same time, it also stipulates that the persons affected should be able to retain control over their data regardless of technological developments ([8] and Recital 15 of [14]) and that the level of data protection must ultimately remain high regardless of the processing technology employed (Recital 10 of [14]).

### Processing personal data in principle

The GDPR defines the sphere of personal data. The definition is broad and includes even pseudonymized data that can be attributed to an individual (known legally as a “natural person”) by drawing on additional information ([22] and Article 4(1) of [14]). For the purposes of scientific research, it is possible—under certain circumstances—to make an exception to the ban on processing sensitive data, such as genetic and medical data (Recital 10, Article 9(1) and Article 9(2)i of [14]). Furthermore, the GDPR requires that confidentiality is ensured on an ongoing basis in the processing of personal data (this is termed “ongoing confidentiality”; Article 32(1)b of [14]). In addition to genetic and clinical data, genetic studies have begun to collect other data, such as information about lifestyle (for example, cigarette consumption), and an increasing quantity and diversity of phenotypic data, thus increasing the identifiability of donors.

In this regard, the so-called “right to be forgotten” is codified in the GDPR (Recital 65 and Article 17 of [14]). As part of this right, the deletion of data and the forgoing of any further processing could be required under certain circumstances, such as when consent has been withdrawn. The further storage of personal data is nonetheless considered legal if, among other reasons, this is necessary for the purpose of scientific research. A necessity exists if the exercising of the right makes achieving the goals of the scientific research impossible or would seriously impair the research (Article 17(3)d of [14]). However, if storage is no longer required for the purpose of scientific research, personal data must be deleted without unreasonable delay following requests made by donors (Article 17(1)a of [14]).

Research relevant to genomic medicine often seeks participation from a large number of patients, and in most instances, the withdrawal of consent by individual patients does not have a substantial influence on the research. However, the more pressing unanswered technical question is how the right to be forgotten can be enforced when data are processed in a public cloud. The localization of individual datasets in global public clouds can give rise to difficulties because of the distributed nature of the data centers of these cloud providers and the likelihood that copies of datasets exist.

The PCAWG consortium has begun collaborating with commercial cloud providers to leverage their enormous computing capacities for the processing of properly consented datasets [12]. However, the use of IT resources from commercial cloud providers, for example, could give rise to additional data processing challenges regarding the responsibilities of the data controller and the data processor and the transfer of personal data to third countries; we discuss these challenges below.

### The responsibility of the data controller and the data processor

In the GDPR, the comprehensive responsibility and accountability of the persons and entities that control data processing are codified for their own processing of personal data and for the processing that is accomplished on their behalf by, for instance, cloud service subproviders (Chapter IV of [14]). The data controllers not only have to take technical and organizational measures in order to be able to prove adherence with the data protection provisions (Article 25(2) of [14]), but also have to take appropriate and effective measures that take into account the nature, scope, context, and purposes of the processing, as well as the risk of compromising the personal rights and freedoms of the persons affected (Article 24(1) of [14]). They must only employ means of data processing that ensure sufficient guarantees for the protection of personal data (Recital 81 and Article 28 of [14]). The controller is responsible for the implementation of all principles of data processing (Article 5(2) of [14]) and, in particular, has the duty of enforcing the rights of affected persons, including—first and foremost—the obligations of conveying information to the affected persons (information obligations) (Article 14 of [14]) and the duty to take appropriate steps to inform further responsible parties of a request for deletion by an affected person (Article 17(2)a, Recital 86 and Article 33 of [14]).

Data processors also have certain obligations. Among other duties, they must provide all necessary information for proving the fulfillment of their obligations to the data controllers in order to demonstrate that they are operating in accordance with the GDPR. Furthermore, they should make it possible for controllers to conduct inspections (Article 28(3)h of [14]). They are also obliged to delete all existing copies of data upon request by the controller (Article 28(3)g of [14]).

However, it is often unclear whether it is the data controller or the data processor who is responsible for the actual implementation of the data processing requirements and guarantees that are applicable according to the GDPR (Articles 31 and 32 of [14]). It is important to note that in the context of genomics studies such as the PCAWG project, commercial cloud providers might cooperate with subproviders without fully revealing the circumstances of their cooperation to data controllers. In such cases, it is possible that the main cloud service provider would itself act as a data controller in the relationship with the subprovider, thus further complicating the clarification of responsibilities.

While the distinction between these responsibilities has some advantages for data controllers, these provisions still take little account of the different relationships that exist between the persons and entities that decide how data should be processed and those who solely carry out data processing on behalf of the controllers, depending on the different partners involved in the cooperation (that is, academic, private, or public cloud providers). If, for example, European researchers acting as data controllers use the cloud services of globally acting commercial cloud providers, it is hardly (if at all) possible for them to fulfill their duties to ensure and monitor data protection standards. Such difficulties in fulfilling these obligations are even more likely to arise in intercontinental collaborations such as the PCAWG project. In the GDPR, both the data controller and the data processor are obliged to make use of solutions to allow the affected person to continue to have the fundamental rights and guarantees that they have in the EU (Recital 114 and Chapter V of [14]). Ongoing growth in the outsourcing of IT infrastructures is making it substantially more difficult for the researchers involved to track and verify global data-processing procedures. Even though researchers may have the best intentions, fulfilling this expectation poses a great challenge to all those involved in research, given the fundamental differences in the understanding and regulation of personal data protection and the rights of persons affected at an international level [13].

### The transfer of personal data to third countries

The GDPR has strict requirements regarding the transfer of personal data to third countries. No specific exception for the transfer of personal data to a third country in the area of scientific research—such as within the context of genomic research consortia like PCAWG—is provided for. In the third country, an adequate level of protection is necessary (Recital 81 and Article 45(1) of [14]). The objective of this requirement is to maintain the protection of the individual as guaranteed by the GDPR, even if the individual’s data are transferred repeatedly (Recital 104 of [14]). In its ruling of 6 October 2015, the European Court of Justice (ECJ; Box 1) declared invalid the decision of the European Commission (EC) that, based on the Safe Harbor Agreement (Box 1) between the US and the EU, the US ensured an adequate level of protection [23, 24]. The ECJ found that the Safe Harbor Agreement does not contain any provision regarding the existence of rules in the US that have been adopted by federal authorities and that intend to limit any interference with the fundamental rights of persons whose data are transferred from the EU to the US. Such interference can be relevant when, for example, state bodies of the US are authorized to access data for legitimate objectives, such as national security. Moreover, the Safe Harbor Agreement does not refer to the existence of effective legal protection against interference of this kind.

In light of the ECJ judgment, the permissibility of data transfer to the US on the basis of other binding agreements or legal instruments, such as binding corporate rules or standard contract clauses (Box 1), has also been called into question [25]. At the beginning of 2016, the EC announced the completion of negotiations with the US over a new data transfer mechanism named the EU–US Privacy Shield [26] (Box 1). After obtaining advice from the Article 29 Data Protection Working Party (an EU advisory body; Box 1) and representatives of the member states, the results of the negotiations were considered to provide a new basis for an “adequacy resolution” by the EC that acknowledges the existence of adequate data protection in the US as a third country. However, the new guidelines have been sharply criticized by data protection experts [27]. An Irish privacy advocacy group (Digital Rights Ireland) has already presented the ECJ with criticisms about the EU–US Privacy Shield; it questioned the adequacy of the EU–US Privacy Shield agreement and argued that it did not sufficiently address the court’s objections to the Safe Harbor Agreement [28]. This emerging criticism calls into question whether there will be constant and legally valid grounds for transatlantic transfers of genetic and genomic research data in the form of an international agreement in the near future.

In the absence of an adequacy decision by the EC, and without sufficient guarantees by means of standard contract clauses or binding corporate regulations, the general derogations (that is, the partial exemptions from the general rules) provided by the GDPR to limit the rights of data subjects in favor of the freedom of research could be drawn on for the benefit of the freedom of research (Recitals 107 and 108 of [14]). Derogations that might possibly be used to facilitate the freedom of research in the fields of genetics and genomics include those relating to the explicit consent of the data subject and the vital interests of the data subject if they are incapable of giving their consent (Article 49(1)a and Article 49(1)f of [14]). If derogations cannot be made use of, then transfers that are not considered large-scale—that is, those that do not occur repeatedly and only affect a limited number of persons— may also be possible in the event of compelling legitimate interests of the data controller (Recital 113 and Article 49(1)g of [14]). The processing of personal data is considered to be large-scale if a large number of data subjects are affected and if the processing would probably constitute a large risk owing, for example, to the sensitivity of the data being processed. The processing is thus also considered to be large-scale if a new technology is extensively used or the technologies used entail a high risk of compromising the rights and freedoms of the persons affected or if their use hinders the enforcement of those rights (Recital 91 of [14]).

It is questionable whether or not the decisive derogation of informed consent could be used in cross-border genetics and genomics research collaborations [29, 30]. A patient’s consent, even if obtained in a dynamic manner, can only confirm their understanding of the scope of data transferability in a very limited way. Also, without knowledge about the specific analysis results of the research, it is often not possible to prove that the vital interest of the person concerned will be affected by the analysis. According to the ECJ’s Safe Harbor Agreement ruling, consent for the transfer of personal data can only form a sound basis for data transfer under the narrow conditions of the transfer not occurring repeatedly, in large quantities, or routinely [31]. Cloud computing in genetics and genomics research may mean that a large quantity of diverse data (including sensitive data) from many thousands of patients is processed at high rates using new IT resources, which would make locating the data and thus the availability of the data for appropriate control and processing more difficult.

## The way forward: addressing regulatory difficulties

We now discuss possible solutions that could overcome regulatory difficulties in order to facilitate the promotion of scientific research and to protect the rights of data subjects. Again, we use the PCAWG project as an example of a genetics and genomics research project that involves patient data, uses cloud computing, and operates within the realm of EU law. We first consider the status and rights of scientific researchers in the EU in relation to the EU’s aim to promote the European Research Area. Second, we weigh up technical solutions that might be employed to protect and fulfill the fundamental rights of the data subjects and the researchers.

### Scientific freedom and the promotion of science under EU law

The specific standardization of scientific freedom as a fundamental EU right in the EU Charter of Fundamental Rights (EUCFR; Box 1) can be understood as an EU position for setting values that must also be taken into account when interpreting fundamental rights, given that the freedom of research cannot be determined with such clarity in the shared constitutional traditions of the member states or the guarantees of the European Convention on Human Rights. The guarantee of the freedom of research should be interpreted broadly, and it is not to be limited except if it violates human dignity or is not compatible with the absolute prohibitions of the EUCFR (Article 1 and Article 3 paragraph 2 of [32]). When considered more closely, limitations to scientific freedom arise primarily from clashing fundamental rights in the EUCFR, including the protection of personal data, and also specific legal provisions that are embedded in other EU laws and in national legislation [33].

The promotion of science is an objective of the EU and is supported by EU treaties (Box 1). According to the Treaty of the EU (TEU), the EU promotes scientific and technological progress (Article 3 III cl. 2 of [34]). The Treaty on the Functioning of the EU (TFEU) defines a European area of research and stipulates that the EU has the objective of strengthening its scientific and technological bases by achieving such a European Research Area. In this area, there should be freedom of movement for researchers, and it should be possible for scientific knowledge and technologies to circulate freely. In creating this area, the EU has the goal of developing competitiveness, including in its industries. To this end, the entire EU must support undertakings (such as projects or enterprises), research centers, and universities in their research and activities for high-quality technological development. The EU must support their efforts to cooperate with each other, particularly so that researchers can cooperate freely across borders and their activities can fully exploit internal market potential. This could be achieved, in particular, by defining common standards and by removing legal and fiscal obstacles to such cooperation (Article 179 of [35]).

The fundamental objective of the EU in its promotion of research is to strengthen its scientific and technological bases, and also to improve competitiveness in the broadest sense, rather than just provide extensive individual freedom of research. The most important instrument for achieving the promotion of research is Europe-wide networking by means of cooperation and coordination [36]. The fundamental right of scientific freedom is primarily a defensive right. No entitlement to benefits for supporting research can be derived for the individual researcher or scientific establishments from the EUCFR (Article 13 of [32], and [37]). The right to freedom of science guaranteed under basic EU law nonetheless also requires that the support of research by EU institutions should be provided in a manner that is as “suited to the needs of science as possible” (page 169 onwards in [36]). Considering that basic and translational genetics and genomics research is increasingly reliant on global cooperation and, in this context, relies on high statistical validity (based on comprehensive datasets), the needs of science must also include international data sharing and global scientific cooperation.

With regard to data protection regulation and the interpretation of the GDPR for scientific research, the objective of creating a European Research Area as stipulated in the TFEU should be taken into account (Article 179 paragraph 1 of [35]). As the GDPR is connected to the EU treaties as secondary law, it must be interpreted in the light of primary law (that is, the EU treaties) in order to be legitimate. Therefore, the requirements of scientific research must be given a weighting similar to that provided for in the EU treaties. In order to achieve this objective, efforts to increase the technological connectivity in Europe are indispensable. However, there remains the question of whether such efforts are compatible with the eligibility conditions for translational research projects such as the PCAWG project, which are required to uphold protection of sensitive data while at the same time ensuring cost-effective solutions and international cooperation.

### Federated clouds for research using sensitive data

A federated cloud setup involves the use of multiple public and/or private cloud resources that are made consistently accessible through the use of joint interoperable protocols, typically to match specific needs (for example, to enable data processors to access otherwise inaccessible datasets; Box 1). Federated clouds can comprise a hybrid cloud solution, which encompasses a combination of on-premises (private) and third-party (public) cloud services. This can involve the combined use of locally managed data centers (which can store valuable, sensitive datasets and can provide cloud computing to restricted communities) and global public cloud computing resources (which can provide vast scalability (Box 1), commensurate with demand, to facilitate particularly computationally intensive research applications; Table 1). The advantages of hybrid and federated cloud models are that they require little centralized planning and can be built using different funding sources, governance structures, and organizational models, as long as agreement exists in terms of standards and interoperable frameworks.

**Table 1:** Benefits of cloud service provision models for storing and computing sensitive data

	Commercial clouds	Private, academic, and community clouds	Federated hybrid clouds
Examples	Amazon Web Services, Google, Microsoft, T-Systems, Seven Bridges Genomics	The Embassy Cloud at the European Bioinformatics Institute of the European Molecular Biology Laboratory [51]	The public–private partnership model of the Helix Nebula Science Cloud Initiative in Europe [38]
Accessibility	Accessible to the public	Locally managed cloud resource; access is limited to a particular community of users	Federated access to locally managed and commercially available off-site cloud resources through the use of joint interoperable protocols
Benefits	Provide on-demand access to competitive large-scale data storage; have the computational and networking resources to scale processing; can readily store non-data privacy critical (parts of) datasets	Provide on-demand access to well-defined and well-managed data storage and computing infrastructure; provide tightly controlled data access, including to data that cannot be transferred across the internet	Federated model helps to differentiate personal data attributability and limit donor re-identification; service brokerage supports the matching of data and processing requirements (for example, certification, data security, interoperability, legislation, and geographic location) against a defined catalogue of services offered by connected providers

A federated cloud model could help to classify and separate personal data and additional information according to its personal attributability and jurisdictional source. The introduction of a corresponding differentiation in data processing could serve to limit the possibility of intentional or unintentional donor re-identification. Storing and processing data or data combinations attributable to persons in separated, confined (federated) clouds could prevent the particular provider from having to disclose data if the specific confined cloud does not fall within the jurisdiction on which the request for disclosure is based.

By confining donor-specific data that might facilitate re-identification to clouds under specific jurisdictions or secure areas (even including areas outside of the cloud for particularly sensitive patient data), the chance of re-identification could be minimized and thus the rights of donors could be better protected. Funders supporting publicly financed research projects also operate on the basis that only one copy of a dataset is saved at a single data center for processing. This is associated not only with security advantages, but also with savings in costs and resources, and is thus compatible with joint processing in a cloud. Such solutions are usually classified as local data centers, although they could also be defined as community clouds in the narrowest sense because the participating academic institutions typically allow for shared on-demand remote computing access to the data in such contexts.

Merging such solutions into a confined part of a federated cloud model—on a European level, for example—would allow for the storage of sensitive research data in the private and community clouds of academic institutions. Combination with a hybrid model would simultaneously allow the involvement of recognized and certified European industry partners to provide the necessary reliable technological infrastructure, and could facilitate the opening up of research to global parties outside the European Research Area. Notably, federating the computing activities in different centers may thereby also enable the involvement of third countries in international collaborations.

Key developments in building a pan-European cloud for science with federated and standardized access to the cloud resources of commercial, public, and academic providers have already been initiated in the context of the Helix Nebula initiative [38], which is a public–private partnership that involves several major European scientific research centers and leading European IT service providers. The efforts of the Helix Nebula Initiative have highlighted the importance of cloud brokerage services and the use of interoperable and standard APIs to allow the efficient and standardized compliant use of federated cloud resources and hybrid clouds. Independent cloud brokers support researchers (and institutions) in the selection, purchase, and access of cloud resources. Individual user requirements—such as the capacity, pricing, certification, data security, interoperability, legislation, or geographic location of data storage and processing services—can be matched against a defined catalogue of services offered by connected providers. Among other advantages, standard APIs support rapid deployment, efficient scale-up, and vendor independence, which could help to address data protection and compliance issues related, for example, to processing restricted datasets.

Furthermore, the European Cloud Initiative has been announced as part of the EC’s Digital Single Market agenda [39]. It is partly funded by the EC as the Initiative will support the implementation of the future EOSC and the complementary European Data Infrastructure. Once established, this digital infrastructure—which is expected to implement a federated and hybrid cloud model—should support researchers in Europe and internationally to securely store, process, share, and re-use data, and should result in a trusted environment across technologies, disciplines, and borders.

One realization that emerged from projects such as the PCAWG project is that while IT processing capacity is necessary, it alone is hardly sufficient to face the emerging challenges. Indeed, one major objective of the PCAWG project and of related approaches for facilitating data sharing—such as the Global Alliance for Genomics and Health (GA4GH) [40]—has been the development of computational protocols that enable interoperability and integration at the level of the software involved: that is, the protocols and interfaces that analysts can employ to share data and process these in a standardized fashion across borders. A federated ecosystem of sharing genomic and clinical data is now being pushed forward by the GA4GH [41]. However, a workable solution must further fulfill data security and data protection requirements, which still frequently differ between jurisdictions, notwithstanding the fact that there is an obvious and general movement in data protection laws toward giving data subjects an active role in the protection of their data, as discussed above [13]. As an example of the pan-European research infrastructure for biological information, ELIXIR has demonstrated that it is possible to strive for the orchestration of the collection, quality control, archiving, and access of large amounts of data in a manner that includes a consideration of regulatory and ethical aspects [42].

## Conclusions

Against the backdrop of European regulations relating to the processing of personal data, the characteristics of new technologies such as cloud computing in translational genetics and genomics research make their international application more difficult. In particular, the definition of personal data, the enforcement of the encryption principle (that is, the anonymization of personal data so that they can only be linked to patients by authorized individuals), and the transfer of data to third countries give rise to difficulties. There are substantial challenges—such as the distribution of responsibilities and obligations between data controllers and data processors—which limit cross-border research collaborations and cooperation between academic and commercial partners, especially in the transatlantic exchange of data. Such limitations prevent new technologies from being applied because individual researchers and research institutions are often not able to fulfill the responsibility that has been assigned to them in relation to protecting personal data and the rights of data subjects (including patients).

International research collaborations and the involvement of public partners in the research also must not, according to the GDPR, result in weaker protection of personal data but must instead be realized through adherence to improved standards in the context of research. At the same time, the development of scientifically and technologically state-of-the-art data-processing technologies is a requirement for the promotion of research that is suitable to the needs of science. Although models of federated and hybrid clouds provide clear advantages over purely public clouds, commercial cloud computing in particular still suffers from the public perception of decreased data privacy and security. Allowing information to be transferred across a network that might be subject to third-party interference or tapping is, for many organizations, an unnecessary security risk. However, storing sensitive patient data and combinations of data in the context of large-scale genetics and genomics research projects—which would only allow the identification of a person in local academic clouds while also involving only approved industry partners in hybrid cloud solutions—might enable compliance with strict data protection regulations, and could foster trustworthy and up-to-date international research.

Facilitating research by providing researchers with access to data via cloud technology creates an impetus for the development of a governance model that uses technological solutions to comply with data protection regulations and at the same time relies on specific standards created by professional organizations. Additionally, if the developed cloud standards establish a framework that enables researchers to make decisions about which projects should use hybrid clouds and which usage patterns are acceptable for translational research, this framework could provide a solution for processing sensitive research data in harmony with data protection regulations. Independent governance that relies on the work of professional organizations also serves to benefit the democratization of bioinformatics research by reducing the dependency of cutting-edge science on institutional IT infrastructure solutions and by giving researchers from universities and research centers equal access to state-of-the-art IT capabilities. Clear terms of access and excellence-driven resource allocation as part of an independent governance would also engender the trust necessary for the management of sensitive data in the cloud in a context where trust is becoming a key challenge for cloud solutions. Such features would likely provide a vital competitive advantage for the European Digital Single Market.

The accelerated expansion of European cloud solutions could ensure a technological basis for researchers to fulfill data-processing requirements. Furthermore, trustworthy European solutions could contribute to member states refraining from taking further measures to restrict the transfer of sensitive data (Recital 10 of [14]) and could also contribute to the free movement of data within the EU without undue interference (Recitals 13, 19, and 166, and Article 1(1) of [14]). A consistent European research infrastructure should qualify Europe for participation in global research while also ensuring that European data protection standards are maintained and the rights of affected persons are guaranteed. The EU-wide “level playing field” for data protection in the form of federated and hybrid cloud models might enable the development of the EOSC in a manner that also permits scientific collaborations with third countries on the basis of data sharing with cloud models that follow legal regulations and ethical standards.

To conclude, a federated and hybrid cloud model could enable internationally established patient rights to be respected worldwide. At the same time, the legal understanding of patient rights, the responsibilities for their realization, and the cultural differences in their implementation could still be taken into account. This could provide the scope to allow differing implementations of subjective rights and the merging of different cloud models into a federated and hybrid solution without hindering progress toward generally increasing the active role of research participants and patient donors in the processing and protection of their data, thus keeping pace with this emerging global trend.

## Box 1 Definitions and explanations of legal and computing terms

**Application programming interface**: a set of definitions, protocols, tools, and clearly defined methods of communication between different software components.

**Article 29 Data Protection Working Party**: a group that was set up in Article 29 of the Data Protection Directive, the data protection regulation of the EU previous to the GDPR. Its membership consisted of the national supervisory authorities of the European Union (EU), the European Data Protection Supervisor, and the European Commission (EC). It will be replaced, according to the General Data Protection Regulation (GDPR), by the European Data Protection Board, which will have a similar membership. Compared to the Article 29 Working Party, which was an advisory body, the European Data Protection Board will have an enhanced status as an independent body of the EU with its own legal personality. See [43] for more information.

**Binding corporate rules**: personal data protection policies that are adhered to by a controller or processor established in the territory of a member state. They regulate transfers or a set of transfers of personal data to a controller or processor in one or more third countries within a group of undertakings, or such transfers within a group of enterprises that are engaged in a joint economic activity (Article 4(20) of [14]).

**Cloud computing**: the storing and large-scale processing of data by multiple users by means of a shared information technology infrastructure in which resources can be requested and released on demand, and by using a remote access connection that is usually established via the Internet (or via a private network in exceptional cases).

**Consent**: any freely given, specific, informed, and unambiguous indication of a data subject’s wishes; this involves the subject providing a statement or a clear affirmative action that signifies their agreement to the processing of their personal data (Article 4(11) of [14]).

**The Court of Justice of the European Union**: the principal judicial institution of the EU. It currently consists of one judge from each member state and eight advocates general. Its function is to ensure the observance of the law. The EC, or another member state, may bring an action before the Court of Justice against a member state on the grounds of a failure to fulfill an obligation under the EU treaties. The European Court of Justice is part of The Court of Justice of the European Union. It is the highest court in the European Union. See [44] for more information.

**Data controller**: a natural or legal person, public authority, agency, or other body that—alone or jointly with others—determines the purposes and means of the processing of personal data (Article 4 (7) of [14]).

**Data processor**: a natural or legal person, public authority, agency, or other body that processes personal data on behalf of the data controller (Article 4(8) of [14]).

**EU Charter of Fundamental Rights**: a human rights catalogue that is legally binding across the EU. It consists of a preamble, 50 articles with individual guarantees, and four articles with general provisions. It serves as a reference document for the fundamental rights that are protected in the EU. See [32] for more information.

**The European Court of Human Rights** (**ECtHR**): an independent judicial body set up within the Council of Europe and established under the 1950 European Convention for the Protection of Human Rights and Fundamental Freedoms. The ECtHR is composed of a number of judges that is equal to the number of contracting parties of the European Convention on Human Rights (ECHR; currently 47). The ECHR gives the ECtHR both contentious and advisory jurisdiction. See [45] for more information.

**EU treaties**: binding agreements approved voluntarily and democratically by all EU member countries. They set out EU objectives, rules for EU institutions, how decisions are made, and the relationship between the EU and its member countries. Under the treaties, EU institutions can adopt legislation that the member countries then implement. The treaties established in the EU are the main source of EU primary law. Secondary sources are legal instruments that are based on the treaties such as unilateral secondary law. See [46, 47] for more information.

**EU**–**US Privacy Shield**: the EU–US Privacy Shield frameworks were designed by the United States (US) Department of Commerce and the EC to provide companies on both sides of the Atlantic Ocean with a mechanism that allows compliance with data protection requirements when transferring personal data from the EU and Switzerland to the US in support of transatlantic commerce. See [48] for more information.

**Federated clouds**: setups that involve the deployment of multiple public and/or private cloud resources that are made consistently accessible through the use of joint interoperable protocols, typically to match specific needs (for example, to enable data processors to access otherwise inaccessible data sets).

**Hybrid clouds**: cloud computing setups that encompass a combination of on-premises (private) and third-party (public) cloud services.

**Interoperability**: the ability of a computer system to run programs from different vendors, and to interact with other computers regardless of the architecture and operating systems used. See [49] for more information.

**Jurisdiction**: the authority of a court or other institution to make decisions or judgments.

**Microbiome**: the community of microorganisms (for example, bacteria, fungi, and viruses) that inhabit a particular environment, and particularly the collection of microorganisms that live in or on the human body.

**Personal data**: any information relating to an identified or identifiable natural person. An identifiable natural person is one who can be identified, directly or indirectly, in particular by reference to an identifier such as a name, an identification number, location data or an online identifier, or by reference to one or more factors specific to their physical, physiological, genetic, mental, economic, cultural, or social identity (Article 4(1) of [14]).

**Petabyte** (**PB**): a multiple of the unit byte, which is used in the quantification of digital information. 1 PB = 10^15^ bytes.

**Pseudonymization**: the processing of personal data in such a manner that the personal data can no longer be attributed to a specific data subject without the use of additional information, provided that such additional information is kept separately and is subject to technical and organizational measures that ensure that the personal data are not attributed to an identified or identifiable natural person (Article 4(5) of [14]).

**Safe Harbor Agreement**: the EU Data Protection Directive prohibited the transfer of personal data to non-EU countries that do not meet the EU “adequacy” standard for privacy protection. In order to bridge differences in data protection approaches and provide a streamlined means for US organizations to comply with the Directive, the US Department of Commerce—in consultation with the EC—developed a Safe Harbor framework to provide the information an organization would need to evaluate and then join the US–EU Safe Harbor program. On 6 October 2015, the European Court of Justice issued a judgment declaring as invalid the EC’s Decision 2000/520/EC of 26 July 2000 “on the adequacy of the protection provided by the safe harbour privacy principles and related frequently asked questions issued by the US Department of Commerce”. See [50] for more information.

**Scalability**: the capability of a computer system or process to handle an increasing amount of work or its potential to be enlarged to accommodate such growth.

**Standard contract clauses**: the EC may set standard contractual clauses for the governance of data processing by a processor under EU or member state law. These clauses are binding on the processor with regard to the controller and set out—among other aspects—the subject matter and duration of the processing, the nature and purpose of the processing, the type of personal data and categories of data subjects, and the obligations and rights of the controller. These clauses are subject to a specific examination procedure according to Article 93(2) and Article 28(7) of [14].

**Third party**: a natural or legal person, public authority, agency, or body other than the data subject, controller, processor, and persons who, under the direct authority of the controller or processor, are authorized to process personal data (Article 4(10) of [14]).

**Use case**: a specific application—for example, the analysis of human genomes with an intended useful scientific outcome—performed by cloud users (in this case scientists).

## Box 2 Advantages and disadvantages of cloud computing

**Advantages**

+ Acceleration of computing processes

+ Rapid scalability upward and downward, commensurate with demand

+ Widespread network access

+ High security safeguards: standardized data security measures allow vast quantities of data to be processed under the same safeguards

+ Reduction of infrastructural and operational costs through resource sharing

**Disadvantages**

Tailoring of cloud service contracts to organization-specific legal or service-level requirements can be difficult

Difficulties in localizing data (some public clouds)

Difficulties in assigning responsibilities between data controllers and data processors

Difficulties in setting common standards of data protection if data can be accessed from different places (that is, different jurisdictions)

Difficulties in comparing cloud service levels or performance (for example, during procurement)

Migration of information technology services and data access into the cloud requires the involvement of teams possessing a specific skill set

Technological differences between cloud providers can create challenges for cross-cloud integration or migration between providers (vendor lock-in)

## Abbreviations

API: Application programming interface
EC: European Commission
ECJ: European Court of Justice
EOSC: European Open Science Cloud
EU: European Union
EUCFR: European Union Charter of Fundamental Rights
GA4GH: Global Alliance for Genomics and Health
GDPR: General Data Protection Regulation
ICGC: International Cancer Genome Consortium
IT: Information Technology
PCAWG: Pan-Cancer Analysis of Whole Genomes
TEU: Treaty of the European Union
TFEU: Treaty on the Functioning of the European Union
US: United States

## References

[1] Stein LD, Knoppers BM, Campbell P, Getz G, Korbel JO (2015). Data analysis: create a cloud commons. Nature.

[2] Hudson TJ, Anderson W, Artez A, Barker AD, Bell C, International Cancer Genome Consortium (2010). International network of cancer genome projects. Nature.

[3] Network CGA (2015). Comprehensive genomic characterization of head and neck squamous cell carcinomas. Nature.

[4] Walter K, Min JL, Huang J, Crooks L, Memari Y, UK10K Consortium (2015). The UK10K project identifies rare variants in health and disease. Nature.

[5] International Cancer Genome Consortium. 2017. http://www.icgc.org. Accessed 17 Feb 2017.

[6] ICGC Data Portal: Pan-Cancer Analysis of Whole Genomes (PCAWG). 2015. https://dcc.icgc.org/pcawg. Accessed 17 Feb 2017.

[7] Genomics England. 2017. http://www.genomicsengland.co.uk. Accessed 17 Feb 2017.

[8] Brors B, Eberhardt W, Eils R, Habermann N, Iakhnin S, Korbel J, et al. White paper. The Applied and Translational Genomics Cloud. 2016. http://www.korbel.embl.de/White-paper-ATCG-Cloud.pdf. Accessed 17 Feb 2017.

[9] Mell P, Grance T. The NIST definition of cloud computing. National Institute of Standard and Technology. 2011. http://nvlpubs.nist.gov/nistpubs/Legacy/SP/nistspecialpublication800-145.pdf. Accessed 17 Feb 2017.

[10] NCI Cancer Genomics Cloud Pilots. National Cancer Informatics. 2013. https://cbiit.nci.nih.gov/ncip/nci-cancer-genomics-cloud-pilots/nci-cloud-initiative. Accessed 2 May 2017.

[11] The European Open Science Cloud for Research pilot project. 2017. https://eoscpilot.eu/. Accessed 2 May 2017.

[12] Butler is a framework for running scientific workflows on public and academic clouds. 2017. https://github.com/llevar/butler. Accessed 2 May 2017.

[13] Molnár-Gábor F. Data protection. In: Grote R, Lachenmann F, Wolfrum R, editors. Max Planck encyclopedia of comparative constitutional law. Oxford University Press. 2017. http://oxcon.ouplaw.com/view/10.1093/law-mpeccol/law-mpeccol-e95. Accessed 2 May 2017.

[14] Regulation (EU) 2016/679 of the European Parliament and of the Council of 27 April 2016 on the protection of natural persons with regard to the processing of personal data and on the free movement of such data, and repealing Directive 95/46/EC (General Data Protection Regulation). OJ L 119. 4 May 2016. p. 1–88. http://eur-lex.europa.eu/legal-content/EN/TXT/?uri=CELEX%3A32016R0679. Accessed 2 May 2017.

[15] Gregorio CG. Protección de datos personales: Europa v Estados Unidos, todo un dilema para América Latina. In: Concha Cantú HA, López-Ayllón S, Tacher Epelstein L, editors. Transparentar al estado: la experiencia Mexicana de acceso a la información; sine nomine et sine soco, Mexico. Universidad Nacional Autónoma de Méxiko; 2004. p. 299.

[16] Constitution of the United States of America. United States Senate. 1992. https://www.senate.gov/civics/constitution_item/constitution.htm. Accessed 2 May 2017.

[17] Katz v. United States, 389 U.S. 347 (1967). US Supreme Court. 1967. https://supreme.justia.com/cases/federal/us/389/347/case.html. Accessed 2 May 2017.

[18] In Re: Directives (redacted) pursuant to section 105B of the Foreign Intelligence Surveillance Act, No. 08-01 (United States Foreign Intelligence Surveillance Court of Review). 2009. https://fas.org/irp/agency/doj/fisa/fiscr082208.pdf. Accessed 2 May 2017.

[19] United States v. Verdugo-Urquidez 494 U.S. 259 (1990). US Supreme Court. 1990. https://supreme.justia.com/cases/federal/us/494/259/. Accessed 2 May 2017.

[20] ACLU v. Clapper, No. 14-42-cv (2015). Court of Appeals (2d Cir). 2015. http://law.justia.com/cases/federal/appellate-courts/ca2/14-42/14-42-2015-10-29.html. Accessed 30 May 2017.

[21] United States v. Ganias, No. 12-240 (2016). Court of Appeals (2d Cir). 2016. http://law.justia.com/cases/federal/appellate-courts/ca2/12-240/12-240-2015-06-29.html. Accessed 30 May 2017.

[22] Alioto TS, Buchhalter I, Derdak S, Hutter B, Eldridge MD, Hovig E (2015). A comprehensive assessment of somatic mutation detection in cancer using whole-genome sequencing. Nat Commun.

[23] 2000/520/EC: Commission Decision of 26 July 2000 pursuant to Directive 95/46/EC of the European Parliament and of the Council on the adequacy of the protection provided by the safe harbour privacy principles and related frequently asked questions issued by the US Department of Commerce (notified under document number C (2000) 2441). OJ L 215, 25 Aug 2000, p. 0007-0047. http://eur-lex.europa.eu/legal-content/en/ALL/?uri=CELEX%3A32000D0520. Accessed 2 May 2017.

[24] Maximilian Schrems v. Data Protection Officer, Judgment of the Court (Grand Chamber) of 6 October 2015, Case C-362/14. ECLI identifier: ECLI:EU:C:2015:650. http://curia.europa.eu/juris/document/document.jsf?text=&docid=169195&pageIndex=0&doclang=EN&mode=lst&dir=&occ=first&part=1&cid=225744. Accessed 2 May 2017.

[25] Communication from the Commission to the European Parliament and the Council on the Transfer of Personal Data from the EU to the United States of America under Directive 95/46/EC following the Judgment by the Court of Justice in Case C-362/14 (Schrems). COM/2015/0566 final. 2015. http://eur-lex.europa.eu/legal-content/EN/TXT/?uri=CELEX%3A52015DC0566. Accessed 2 May 2017.

[26] Commission Implementing Decision (EU) 2016/1250 of 12 July 2016 pursuant to Directive 95/46/EC of the European Parliament and of the Council on the adequacy of the protection provided by the EU-US Privacy Shield (notified under document C(2016) 4176). OJ L 207, 1.8.2016, p.1-112. http://eur-lex.europa.eu/legal-content/EN/TXT/?uri=uriserv:OJ.L_.2016.207.01.0001.01.ENG&toc=OJ:L:2016:207:FULL. Accessed 3 May 2017.

[27] Weichert T. EU–US Privacy Shield: is transatlantic data transfer compatible with fundamental rights? Zeitschrift für Datenschutz. 2016;209–17 (in German).

[28] DRI challenges independence of Ireland’s Data Protection Authority. Digital Rights Ireland. 2016. https://www.digitalrights.ie/dri-challenges-idependence-of-irelands-data-protection-commissioner/. Accessed 17 Feb 2017.

[29] Molnár-Gábor F, Korbel, JO. Processing of patient data in the cloud: freedom of translational research and data protection in Europe. Zeitschrift für Datenschutz. 2016;274–81. (in German).

[30] Dove S, Joly J, Tassé AM (2015). Public Population Project in Genomics and Society (P3G) International Steering Committee, International Cancer Genome Consortium (ICGC) Ethics and Policy Committee, Knoppers BM. Genomic cloud computing: legal and ethical points to consider. Eur J Hum Genet.

[31] Position paper of the independent data protection authorities of the Federation and the States (Data Protection Conference), Safe-Harbor – Update (30 Oktober 2015), Nr. 9. 2015. https://www.datenschutz.hessen.de/ft-europa.htm#entry4521. Accessed 17 Feb 2017 (in German).

[32] Charter of fundamental rights of the European Union. Official Journal of the European Communties. OJ C 326, 26.10.2012, p. 391–407. http://www.europarl.europa.eu/charter/pdf/text_en.pdf. Accessed 2 May 2017.

[33] The right to personal data protection is codified in Art. 8 para. 1 EUCFR. Eikenberg H. Art. 179 TFEU. In: Grabitz E, Hilf M, Nettesheim M, editors. The Law of the European Union. Munich: Beck; 2015, recital 57 (in German).

[34] Consolidated version of the Treaty on European Union. OJ C 326, 26.10.2012, p. 13–390. 2012. http://eur-lex.europa.eu/legal-content/EN/TXT/?uri=CELEX%3A12012M%2FTXT. Accessed 2 May 2017.

[35] Consolidated version of the Treaty on the Functioning of the European Union. OJ C 326, 26.10.2012, p. 47–390. 2012. http://eur-lex.europa.eu/legal-content/en/ALL/?uri=CELEX%3A12012E%2FTXT. Accessed 2 May 2017.

[36] Ohlenschläger F. Research Funding Law of the European Union. Verlag Österreich. 2012. (in German).

[37] Lindner JF. The Europeanisation of science law. Mohr Siebeck; 2009, p. 88 (in German).

[38] Helix Nebula. 2017. http://helix-nebula.eu. Accessed 17 Feb 2017.

[39] European Cloud Initiative to give Europe a global lead in the data-driven economy. European Commission. 2016. http://europa.eu/rapid/press-release_IP-16-1408_en.pdf. Accessed 17 Feb 2017.

[40] The Global Alliance for Genomics and Health. 2017. http://genomicsandhealth.org. Accessed 17 Feb 2017.

[41] GENOMICS (2016). A federated ecosystem for sharing genomic, clinical data. Science.

[42] ELIXIR. 2017. https://www.elixir-europe.org. Accessed 17 Feb 2017.

[43] Article 29 Working Party. European Commission. 2016. http://ec.europa.eu/newsroom/just/item-detail.cfm?item_id=50083. Accessed 2 May 2017.

[44] Court of Justice: presentation. 2010. http://curia.europa.eu/jcms/jcms/Jo2_7024/en/. Accessed 2 May 2017.

[45] European Court of Human Rights. 2017. http://www.echr.coe.int/Pages/home.aspx?p=home. Accessed 2 May 2017.

[46] EU Treaties. European Union. 2017. https://europa.eu/european-union/law/treaties_en. Accessed 2 May 2017.

[47] Sources of European Union law. European Union. 2010. http://eur-lex.europa.eu/legal-content/EN/TXT/?uri=URISERV%3Al14534. Accessed 2 May 2017.

[48] Privacy Shield Framework. 2017. https://www.privacyshield.gov/welcome. Accessed 2 May 2017.

[49] Interoperability. Business Dictionary. 2017. http://www.businessdictionary.com/definition/interoperability.html. Accessed 2 May 2017.

[50] Welcome to the U.S.–EU Safe Harbor. Export.gov. 2017. http://2016.export.gov/safeharbor/eu/eg_main_018365.asp. Accessed 2 May 2017.

[51] Services. EMBL-EBI. 2017. http://www.ebi.ac.uk/services. Accessed 30 May 2017.

